# Lipocalin 2 regulates intestine bacterial survival by interplaying with siderophore in a weaned piglet model of *Escherichia coli* infection

**DOI:** 10.18632/oncotarget.18528

**Published:** 2017-06-16

**Authors:** Bing-Xiu Guo, Qian-Qian Wang, Jia-Hui Li, Zhen-Shun Gan, Xiao-Feng Zhang, Yi-Zhen Wang, Hua-Hua Du

**Affiliations:** ^1^ College of Animal Science, Zhejiang University, Hangzhou, 310058, China; ^2^ Key Laboratory of Animal Nutrition and Feed Science, Eastern of China, Ministry of Agriculture, Zhejiang University, Hangzhou, 310058, China; ^3^ Institute of Animal Husbandry and Veterinary Science, Zhejiang Academy of Agricultural Sciences, Hangzhou, 310021, China

**Keywords:** lipocalin 2, siderophore, Escherichia coli K88, iron sequestration, intestinal infection

## Abstract

Iron is an essential nutrient that facilitates cell proliferation and growth, which plays a pivotal role in modulating the battle for survival between mammalian hosts and their pathogens. Pathogenic bacteria secrete siderophores to acquire iron from the host. However, lipocalin 2 (Lcn2), a siderophore-binding antimicrobial protein, binds to siderophores to prevent bacterial uptake of iron, which is critical for the control of systemic infection with *Escherichia coli* (*E. coli*). But few studies focus on the anti-infective response of Lcn2 in the intestines by inhibiting bacterial proliferation based on microbial iron metabolism. In this study, we showed that iron was sequestrated within cells in a piglet model of *E. coli* K88 infection. Siderophores was produced following *E. coli* K88 infection and siderophore-related genes expression was upregulated in iron-deficiency environment *in vitro*. Meanwhile, we found that Lcn2 expression was rapidly and robustly induced in jejunum by *E. coli* K88 infection and could be stimulated by IL-17 and IL-22. Furthermore, both Lcn2 induced in epithelial cells IPEC-1 and added exogenously as a recombinant protein could inhibit the growth of *E. coli*. We can conclude that Lcn2 is a crucial component of mucosal immune defense against intestinal infection with *E. coli* K88.

## INTRODUCTION

Iron is an essential nutrient for most organisms, with crucial functions in many cellular processes. It serves as a cofactor for enzymes in the mitochondrial respiration chain, citric acid cycle or DNA synthesis, as well as being the central molecule for binding and transporting oxygen by haemoglobin and myoglobin [1]. Therefore, iron is necessary for cell replication, metabolism and growth. Both the hosts and the pathogens depend on and compete for iron for their proliferation and biologic functions. Although iron is the second most abundant element in the earth’s crust, iron lies at the centre of an eons-long battle between hosts and their pathogens [2]. The majority of circulating iron is bond to host proteins, resulting in low free-iron availability (10^–24^ M), which functions as a “nutritional immunity” mechanism for controlling bacterial growth [3].

Enterotoxigenic *Escherichia coli* (*E. coli*) are the leading bacteria that cause diarrhea in humans, and the main cause of neonatal and post-weaning diarrhea in livestock animals [4]. It can sense the low iron levels in the host (for example, hypoferremia of inflammation) as an environmental cue to trigger the synthesis of virulence factors [5]. In order to acquire the iron necessary for growth in the iron-limiting conditions, *E. coli* secretes the siderophore enterobactin, which is a prototypical catecholate siderophore with high affinity for iron [6, 7]. Iron acquisition by siderophores plays a significant role in extraintestinal pathogenic *E. coli* virulence [8].

To counter the iron-scavenging effects of enterobactin, neutrophils and host mucosal cells secrete lipocalin 2 (Lcn2), also known as siderocalin or neutrophil gelatinase-associated lipocalin (NGAL) [9]. It can bind enterobactin either in its iron-laden or iron-free forms to disrupt bacterial iron acquisition [10]. Lcn2 is critical for host defense, which enhances inflammation *in vitro* and *in vivo* in response to enterobactin [11, 12]. In this way, Lcn2 may tailor anti-infective response by inhibiting bacterial proliferation based on microbial iron metabolism.

In the current study, we examined the role of Lcn2 in intestinal defense against bacterial infection and the mechanism of its regulation in a piglet model of *E. coli* K88 infection. We showed that *E. coli* K88 infection sequestrated iron within cells and iron-depleted environments induced more siderophores produced by *E. coli* K88 to improve iron acquisition. Meanwhile, Lcn2 was robustly upregulated by IL-17/IL-22 after *E. coli* K88 challenge and limited bacterial growth by interplaying with its siderophore enterobactin.

## RESULTS

### Establishment of a piglet model of *E. coli* K88 challenge

**Figure 1:**
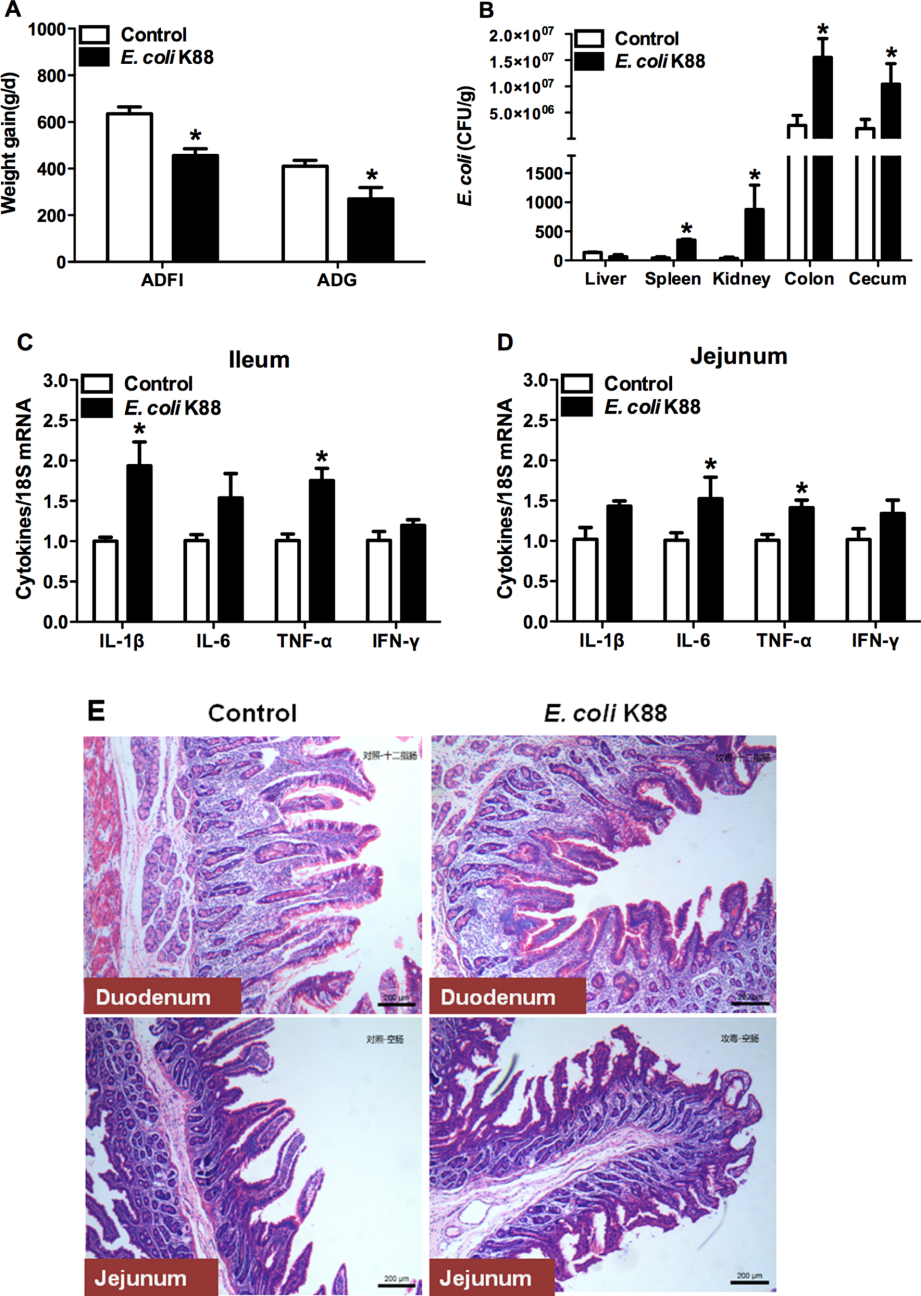
Establishment of a piglet model of E.coli K88 challenge Effects of challenge on average daily feed intake (ADFI), average daily gain (ADG) (**A**), *E. coli* populations in tissues (**B**), transcription levels of inflammatory cytokines in ileum (**C**) and jejunum (**D**) and morphology of duodenum and jejunum of piglets (**E**). mRNA levels of IL-1b, IL-6, TNF-a and IFN-g were determined by qRT-PCR. 18S rRNA was used as the housekeeping gene. mRNA expression ratio was normalized to the mean value of control group of 1. Error bars indicate SD. *p < 0.05.

In order to test the role of Lcn2 *in vivo*, we need to develop a bacterial-challenge model. To do this, each weaned piglet was inoculated orally with 10^10^ CFU of *E. coli* K88. After *E. coli* K88 infection, challenged piglets had lower average daily feed intake (*p* < 0.05) as well as poorer average daily gain (*p* < 0.05) than control piglets (Figure 1A). What’s more, total viable counts of *E. coli* in spleen, kidney, colon and cecum tissues were increased significantly (*p* < 0.05) in challenged piglets (Figure 1B). Meanwhile, mRNA expressions of inflammatory cytokines were increased to varying degrees in ileum (Figure 1C) and jejunum (Figure 1D) of challenged piglets. The secretions of cytokines IL-1β and TNF-α in the ileum, and TNF-α in the jejunum were higher in challenged piglets than in control piglets (*p* < 0.05).

In order to certify the tissue damages caused by *E. coli* K88 challenge, Hematoxylin and eosin (HE) staining for intestines was performed. As expected, villus height was obviously lower in duodenum and jejunum of challenged piglets than in control piglets (Figure 1E). After challenge, the edge of villus was unclear. Goblet cells secretion was increased in the epithelium of duodenum and villus was fractured in the jejunum. In general, bacterial challenge greatly affected villus morphology and the damages were evident.

### Iron distribution in piglets after *E. coli* K88 challenge

**Figure 2:**
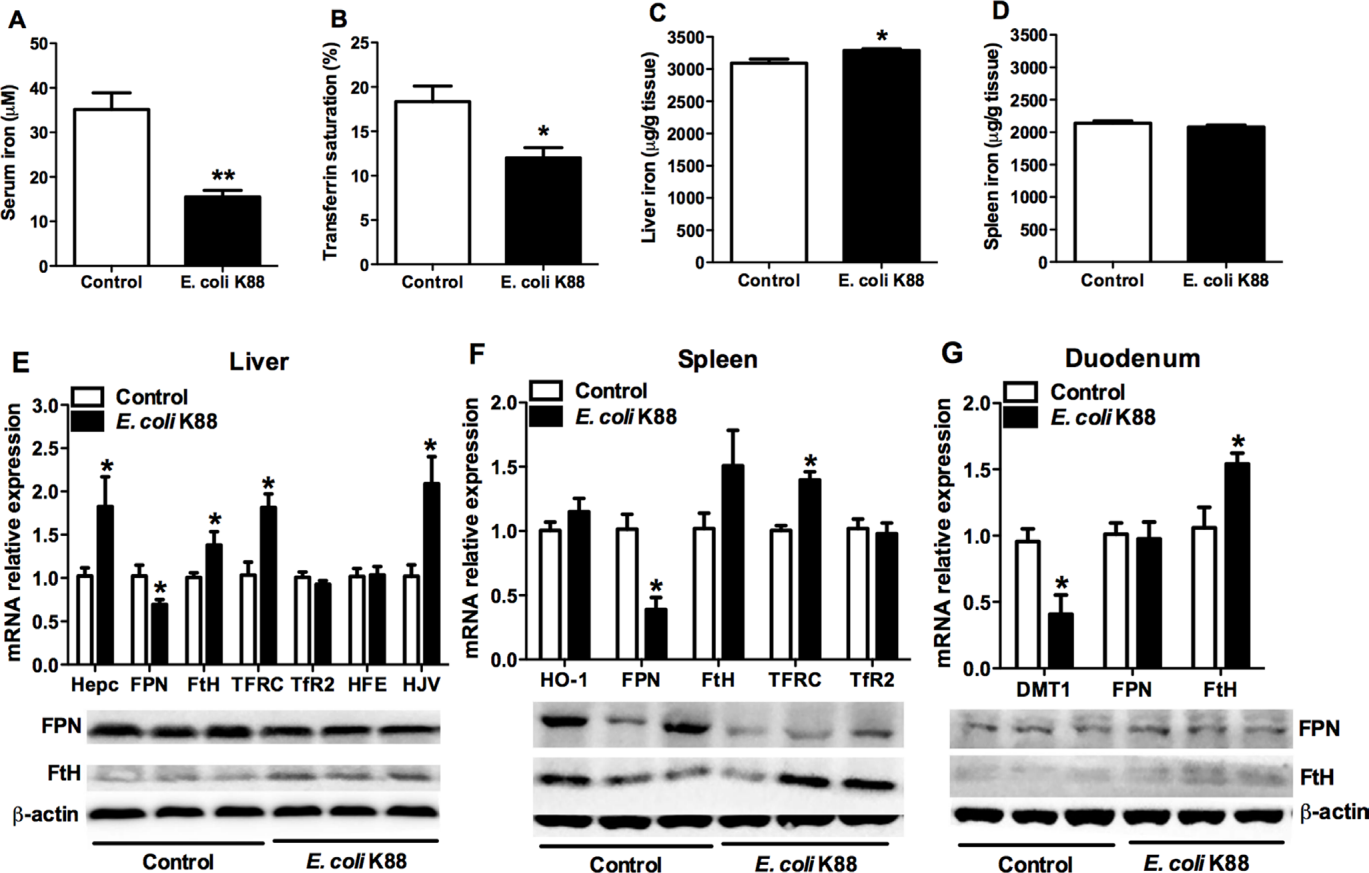
The alteration of serum and tissue iron concentration in *E. coli* K88-challenged piglets with decreased FPN mRNA and protein levels. Control and challenged piglets were analyzed for circulating serum iron levels (**A**), transferrin saturation (**B**), and nonheme iron content in liver (**C**) and spleen (**D**). The same piglets were analyzed for mRNA and protein expressions of iron metabolism related genes in liver (**E**), spleen (**F**) and duodenum (**G**). Error bars indicate SD. *p < 0.05, **p < 0.01.

We next evaluated how the challenge of *E. coli* K88 affected iron distribution and its regulation. After challenge, serum iron and transferrin saturation were both decreased significantly (*p* < 0.05, Figure 2A and 2B). On the contrary, the deposition of nonheme iron in the liver was increased (*p* < 0.05, Figure 2C) whereas no difference of that was found in the spleen (Figure 2D). Furthermore, liver, spleen and duodenum tissues were selected to determine the expression of iron metabolism-related genes. The transcripts of iron regulators including hepcidin, hemojuvelin (HJV) and transferrin receptor (TFRC) were all significantly increased (*p* < 0.05) in liver (Figure 2E, top panel) and spleen (Figure 2F, top panel). Meanwhile, mRNA expressions of iron storage related gene ferritin-H (FtH) were also increased in all detected tissues (Figure 2E–2G, top panel). However, the levels of iron transportation-related genes mRNA expressions including iron exporter ferroportin (FPN, Figure 2E and 2F, top panel) and iron importer divalent metal transporter 1 (DMT1, Figure 2G, top panel) were both decreased significantly (*p* < 0.05). In addition, protein levels of FPN were both decreased in liver and spleen (Figure 2E and 2F, bottom panel), while those of FtH were all increased in these tissues (Figure 2E–2G, bottom panel), which was consistent with mRNA levels. All above results suggested that *E. coli* K88 challenge caused iron sequestration in the reticuloendothelial system of tissues and decreased the circulations of iron.

### Regulation of siderophores synthesis in iron-deficiency environment

**Figure 3:**
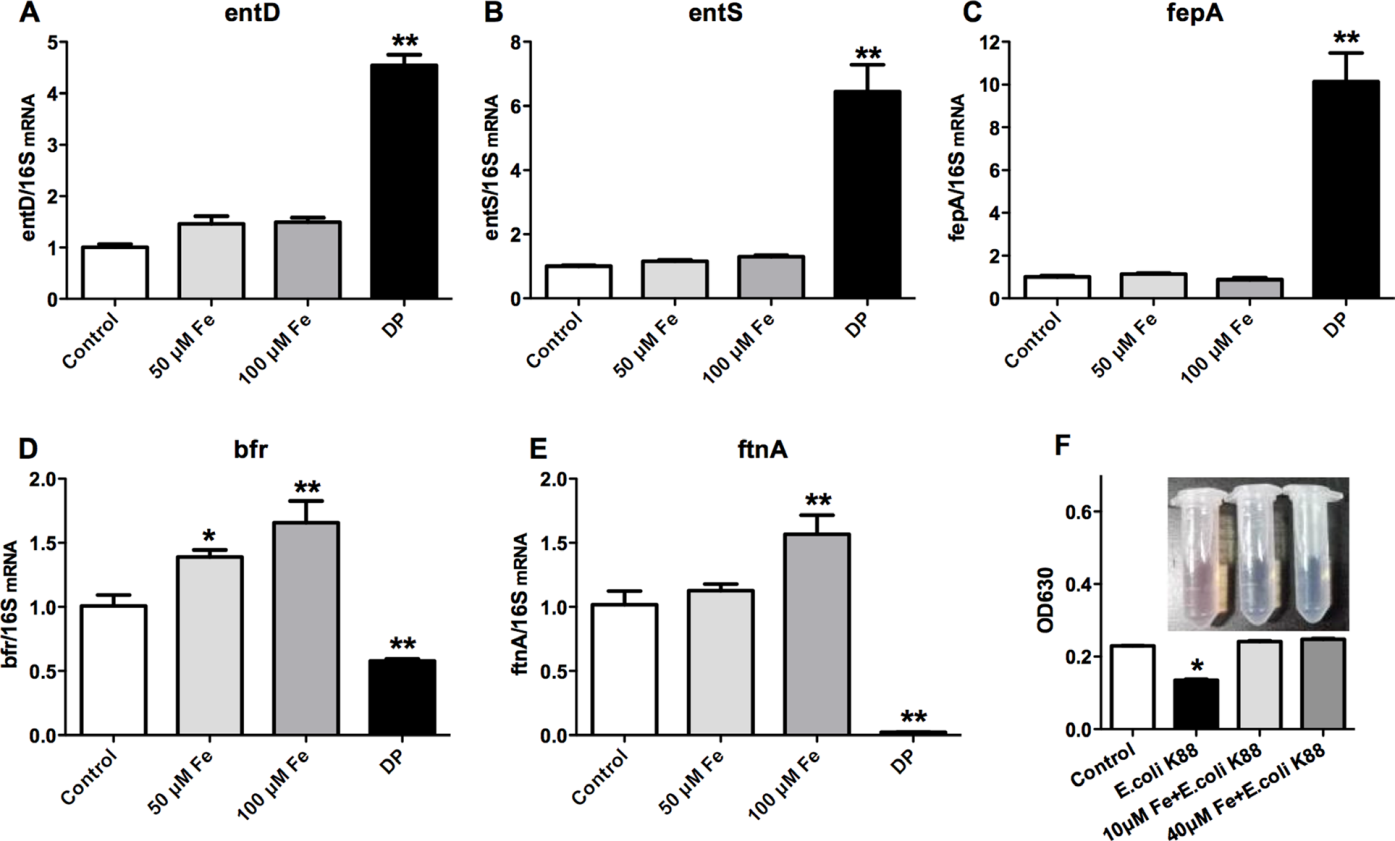
Effects of low iron concentration on the expression of bacterial iron metabolism-related genes and siderophores *E. coli* K12 grown in LB media at 37°C separately treated with 50 mM Fe^3+^ (FeCl_3_), 100 mM Fe^3+^ (FeCl_3_) and 200 mM 2,2-Dipyridyl (DP), then the expressions of bacterial siderophore-related genes entD (**A**), entS (**B**), fepA (**C**) and iron metabolism-related protein bfr (**D**) and ftnA(**E**) were analyzed. *E. coli* K88 grown in M9 media at 37°C separately treated with 10 mM Fe^3+^ (FeCl_3_), 40 mM Fe^3+^ (FeCl_3_), then the secretion level of bacterial siderophores was analyzed (**F**). Error bars indicate SD. *p < 0.05, **p < 0.01.

As *E. coli* K88 challenge decreased the circulations of iron, we next investigated whether the deficiency of iron would affect bacterial growth. Iron acquisition by siderophores plays a significant role in extraintestinal pathogenic *E. coli* growth and virulence (Cusumano et al., 2010). Since bacterial iron metabolism is best understood in *E. coli* K12, this organism was the focus of this study. The mRNA analysis showed that addition of Fe^3+^ (FeCl_3_) did not affect the expression of siderophores synthesis gene entD (Figure 3A), siderophores exporting gene entS (Figure 3B), and siderophores receptor gene fepA (Figure 3C) of *E. coli* K12 (*p* > 0.05). However, the expressions of siderophore-related genes were significantly increased (*p* < 0.01) after treatment with 2,2-Dipyridyl, which can chelate iron and induce iron deficiency. Meanwhile, iron supplementation significantly increased (*p* < 0.01) the mRNA expression of bacterial iron storage protein bfr (Figure 3D) and ftnA (Figure 3E). But after treatment with 2,2-Dipyridyl, transcripts of bfr and ftnA genes were both significantly reduced (*p* < 0.01). Changes of bacterial iron metabolism-related genes expression would directly affect the ability of bacteria to synthesize siderophores. We therefore detected the synthesized siderophores under different iron concentrations. CAS assay was employed and the absorbance of the mixture was measured at OD_630_ for indicating the value of siderophore secretion. The results showed that there was no difference between additions of 10 µM and 40 µM Fe^3+^. However, the OD_630_ absorbance of no treatment was significantly lower (*p* < 0.05), which suggested that *E. coli* K88 secreted siderophores when lived in environment with low iron concentration. All above results indicated that bacteria drastically upregulated expression of siderophores to acquire this essential metal during conditions of iron scarcity.

### Lcn2 production in company with up-regulation of IL-22 and IL-17

**Figure 4:**
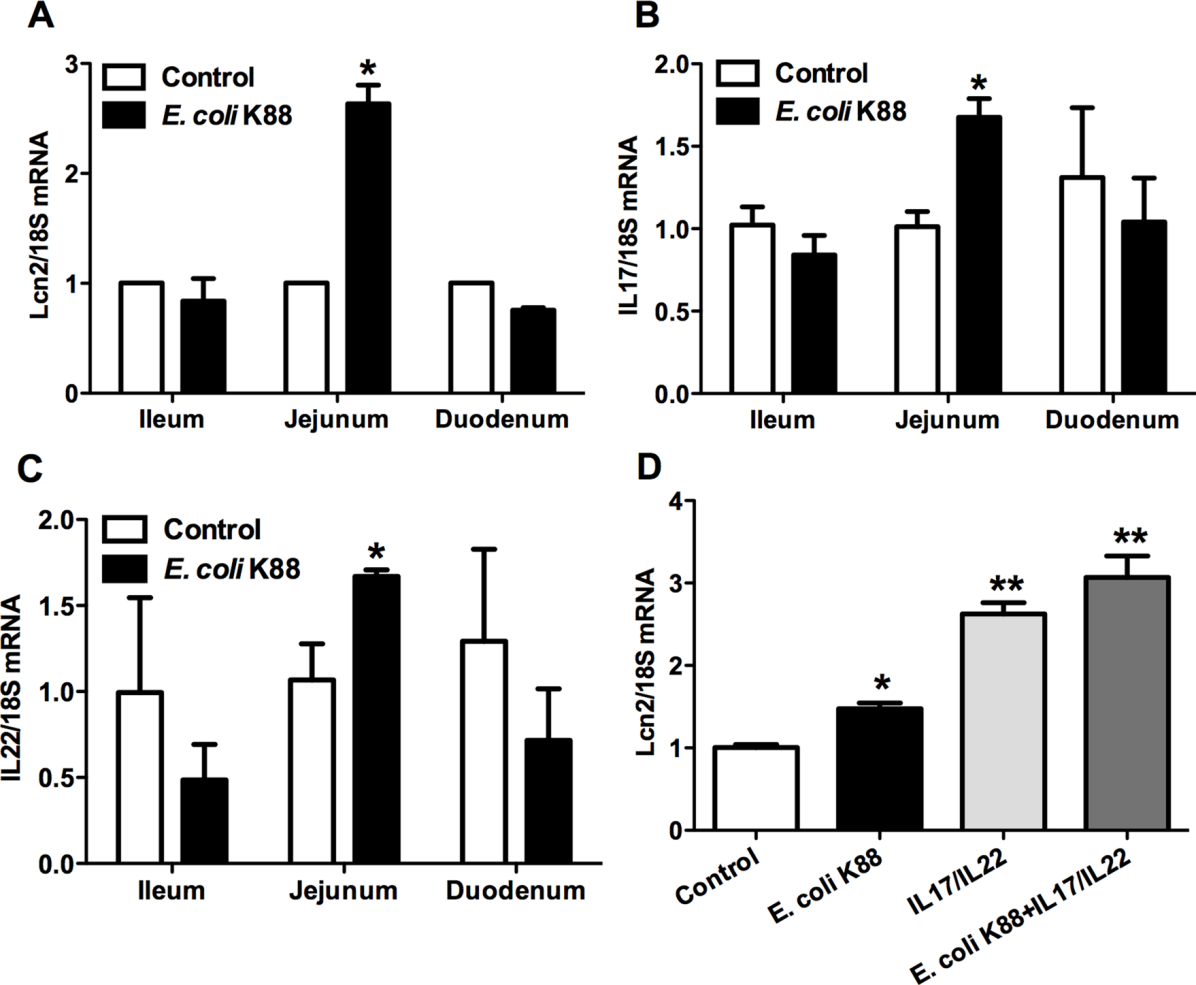
Effects of *E. coli* K88 challenge and IL-17/IL-22 on the Lcn2 expression Control and challenged piglets were analyzed for Lcn2 (**A**), IL-17 (**B**) and IL-22 (**C**) mRNA levels in ileum, jejunum and duodenum. Epithelial cells IPEC-1 were analyzed for effects of IL-17 and IL-22 stimulation on the expression levels of Lcn2 mRNA (**D**). Error bars indicate SD. *p < 0.05, **p < 0.01.

As Lcn2 is an iron-trafficking protein that binds iron through association with a bacterial siderophore, we then detected the expression of Lcn2 in iron deficient intestines caused by *E. coli* K88 challenge. The transcript of Lcn2 was increased significantly (*p* < 0.05) in jejunum of challenged piglets, but remained similar level in ileum and duodenum (Figure 4A). Meanwhile, we found that cytokines interleukin-17 (IL-17) and IL-22 presented a similar expression pattern. That is, challenge did not affect the secretion of IL-17 and IL-22 in ileum and duodenum (*p* > 0.05), but significantly increased (*p* < 0.05) them in jejunum (Figure 4B and 4C). This similar expression pattern made us to suppose that whether there exists a relationship between Lcn2 and IL-17/IL-22. In order to certify this hypothesis, we further detected the mRNA expression of Lcn2 when stimulated by IL-17/IL-22 or *E. coli* K88 infection in IPEC-1 cells. As shown in Figure 4D, IL-17/IL-22 (*p* < 0.01) or challenge (*p* < 0.05) individually induced Lcn2 secretion and the combination of IL-17/IL-22 and challenge induced much more Lcn2 secretion. It suggested that IL-17 and IL-22 were crucial regulators to induce intestinal Lcn2 expression.

### Bacteriostatic effects of Lcn2 on *E. coli* survival

**Figure 5:**
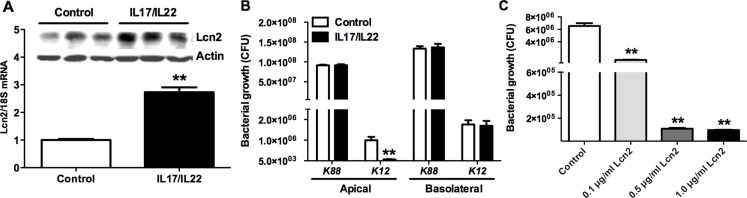
The bacteriostatic effects of Lcn2 Lcn2 expression induced by IL-17 and IL-22 in transwell culturing monolayer epithelial cells IPEC-1(**A**). Effects of apical and basolateral cell culture supernatants of transwell culturing monolayer epithelial cells IPEC-1 on *E. coli* K12 and *E. coli* K88 proliferation by measuring OD_600_ absorbance (**B**). Effects of additional 0.1 µg/ml, 0.5 µg/ml, and 1 µg/ml recombinant Lcn2 on *E. coli* K88 proliferation (**C**). Error bars indicate SD. *p < 0.05, **p < 0.01.

To confirm the bacteriostatic effects of Lcn2 in challenged intestine, we established the transwell culturing monolayer epithelial cells IPEC-1 model of Lcn2 expression induced by IL-17 and IL-22. The mRNA and protein levels of Lcn2 were much higher (*p* < 0.01) than controls, which indicated that the model was successfully established (Figure 5A). Then we used the apical and basolateral culture supernatants of cells treated with *E. coli* K12 to determine the main site of Lcn2 secretion in the intestines. The results showed that apical cell culture supernatants, instead of basolateral supernatants, significantly inhibited (*p* < 0.01) *E. coli* K12 proliferation (Figure 5B). But there was no bacteriostatic activity for cell culture supernatants treated with *E. coli* K88. We further determined whether endogenously produced Lcn2 is insufficient to take effect on bacteriostasis. Then we treated *E. coli* K88 with additional 0.1 µg/ml, 0.5 µg/ml or 1 µg/ml recombinant Lcn2, respectively. With increased addition of Lcn2, *E. coli* K88 proliferation was significantly (*p* < 0.01) inhibited in a dose-dependent manner (Figure 5C). It suggested that Lcn2 had bacteriostatic activity and might play an important role in *E. coli* K88 challenge piglets.

## DISCUSSION

Weaning is a particularly susceptible period, as the removal of maternal immune factors coincides with the increased exposure to pathogens. *E. coli* K88 is a common causative agent of diarrhoea in this period as it releases a kind of choleragen-like enterotoxin, which adheres to intestinal epithelial cells and induces excessive secretion of electrolytes into the lumen of intestine [13]. *E. coli* infection always promotes the innate immune response in small intestines, inculding toll-like receptors proteins, proinfalmmatory cytokines and autophagy [14, 15]. The piglet has been used extensively as a model for infant *E. coli* infection [16]. In this study, a decreased growth performance was found after *E. coli* infection with much lower average daily feed intake and average daily gain. The total viable counts of *E. coli* were expected increased in tissues of challenged piglets. In addition, duodenal and jejunal intestinal sections demonstrated an obvious atrophy after infection, while inflammatory cytokines were increased in ileum and jejunum, which supposed to activate mucosal immunity [4]. The results showed that the piglets were mildly infected by *E. coli* K88, which indicated that the challenge model had been successfully established.

Among all the nutrients, iron plays a particularly crucial role in mediating host-pathogen interactions. In animals, evidence for this comes from the diversity of their methods of iron sequestration [17]. In this study, we found that *E. coli* K88 infection redistributed iron deposition in liver of piglets and decreased the serum iron, which supposed to restrict iron available to the pathogen *E. coli* K88. We also examined whether variants in host genes may influence these outcomes. Liver-produced peptide hepcidin controls the main inflows of iron into plasma by several approaches, such as regulating the absorption of intestinal dietary iron, the release of hemoglobin iron recycled by macrophages and the transfer from iron-storing hepatocytes [18]. The finding of increased hepcidin mRNA in challenged piglets was consistent with previous studies that infection and inflammation could immediately up-regulate hepcidin expression, thereupon regulate iron circulation. FPN is the direct target of hepcidin, acting as the iron exporter on macrophages and the basolateral membrane of duodenal enterocytes [19]. We found that FPN was significantly decreased in major iron metabolic organs such as liver, spleen and duodenum, which is consistent with the alteration of hepcidin expression. The *E. coli* K88 challenge increased the expression of TFRC in spleen and liver, indicating that the serum iron was transferring into parenchymal organs. DMT1 located at the plasma membrane transports Fe^2+^ into the cell cytoplasm [20], mainly serves as absorbing dietary iron in duodenum. The decline of DMT1 expression in challenged piglets attenuated intestinal iron absorption thereupon limited the amount of serum iron. Ferritin serves as the depot site for iron storage including L- and H-subunits, the increase of cellular iron enhances the expression of FtH [21]. The increased expression of FtH in liver indicated the increased cellular iron depositing. Taken together, the alterations of iron metabolism-related genes and proteins expressions were consistent with the changes of serum iron content and tissue iron deposition. Host sequestered iron within phagocytes and enterocytes leading to hypoferremia through hepcidin-dependent mechanism under control of *E. coli* K88 challenge.

On the other hands, bacteria require iron to establish infections, propagate pathogenesis and resist host defences. The strongest evidence comes from the high degree of genomic investment in systems for iron acquisition, which permits them to be effective even in highly iron-depleted environments [17]. In our study, the iron-withholding strategies restricted the supply of iron to *E. coli* K88*. E. coli* had evoked the strategy to acquire iron by regulating genomic expression. Enterobactin, a catecholate siderophore released by *E. coli*, has high affinity with iron (K_d_ of 10^–35M^ at physiologic pH), which allows it to capture iron from iron-binding proteins of host such as ferritin and transferrin [22]. Our results indicated that under condition of low iron environment, the expression of siderophore-related genes entD, entS and fepA were increased, as well as the iron acquisition of bacteria was improved. Addition of 50 µM Fe^3+^ and 100 μM Fe^3+^ showed no difference in the expression of siderophore-related genes. One probable reason was that the LB media in the control group contained traces of iron (5 μM), which was enough to meet the bacteria’s requirement for survival. However, the transcriptions of iron storage-related genes bfr and ftnA were just the opposite, which increased with addition of iron and decreased with iron deficiency. It suggested that iron deficiency environment could stimulate the production of enterobactin. The hypothesis was proved by that *E. coli* K88 produced enterobactin after treatment with 2,2-Dipyridyl instead of addition of 10 μM Fe^3+^ or 40 μM Fe^3+^. In other words, when environmental iron was deprived by host, the *E. coli* K88 would secrete siderophores to compete for iron against host defense.

As a countermeasure, host secretes Lcn2 to compete with bacteria for iron. Lcn2 is able to bind both ferric and ferrous enterobactin, depleting the iron-scavenging siderophore enterobactin from bacteria and preventing *E. coli* from uptake of enterobactin-bound iron [12]. In our study, Lcn2 was mainly produced in liver of either challenged or unchallenged piglets, which indicated that liver is extremely crucial in the innate immune system (data not shown). Since bacterial infection first occurred in the gastrointestinal tract, we focused on the expression of Lcn2 in various intestinal segments. The results showed that Lcn2 mRNA level was increased significantly in jejunum, but unaltered in ileum and duodenum. Meanwhile, transcriptions of IL-22 and IL-17 were merely increased in jejunum too, which was consistent with the alteration of Lcn2 in jejunum. Similar expression distribution raised a doubt whether there was a certain link between Lcn2 and IL-17/IL-22. IL-17 and IL-22 are secreted by Th17 cells, promote neutrophils recruiting and prevents pathogens from disseminating, as well as stimulate epithelial proliferation and protects the intestinal barrier function during infection [23, 24]. Furthermore, IL-17 and IL-22 could induce marked increase of antimicrobial proteins including Lcn2 protein, the Lcn2 expression was dramatically decreased in intestine of IL-22^−/−^ mice [25]. Here, IL-22 and IL-17 co-treatment induced high expression of Lcn2 in intestinal epithelium cells IPEC-1, which confirmed that elevated Lcn2 expression in *E. coli* K88-challenged piglets was related to high secretion of IL-17/IL-22.

Next we took advantage of the transwell culturing monolayer epithelial cells IPEC-1 model of Lcn2 expression induced by IL-17 and IL-22 to determine the bacteriostatic effects of Lcn2 in challenged intestine. The growth of *E. coli* K12 was inhibited by apical cells culture supernatants, but not basolateral cells culture supernatants. It suggested that the secretion of Lcn2 in IL-17/IL-22-stimulated intestinal epithelial cells occurred from the apical surfaces of cells. Considering that Lcn2 specifically binds enterobactin, the apical secretion of Lcn2 can affect *E. coli* K88 growth within the intestinal lumen. However, endogenous Lcn2 had no bacteriostatic effect on *E. coli* K88 growth. It was presumable that IL-17/IL-22 induced Lcn2 was inadequate to take effect on restraining *E. coli* K88 proliferation. Therefore, we treated *E. coli* K88 with additional recombinant Lcn2 in different concentrations to prove this assumption. The result showed that the growth of *E. coli* K88 was gradually inhibited with the increase of additional recombinant Lcn2. It indicated that either endogenous Lcn2 or exogenous Lcn2 had bacteriostatic effects on *E. coli* K88 growth. The inhibition demonstrated that Lcn2 acted as part of the first line of defence against invading microbes by chelating bacterial siderophores with high affinity (0.4 nM ) [26], and depriving them of iron.

In summary, our study demonstrated that Lcn2 mediated an innate immune response to *E. coli* K88 infection by interplaying with enterobactin to compete iron. Our data in the piglet model indicated that the *E. coli* K88 infection resulted in iron sequestrated within cells and iron-depleted environments induced *E. coli* K88 to produce more siderophores to facilitate iron acquisition. Meanwhile, upon encountering bacteria, innate immune cells produced and secreted Lcn2 to bind siderophore to deprive bacteria of their iron-uptake ability, which could limit the growth of bacteria.

## MATERIALS AND METHODS

### Bacterial strains

Enterotoxigenic *Escherichia coli* K88 C83907 (*E. coli* K88) and *E. coli* K12 MG1655 (*E. coli* K12) were purchased from China Institute of Veterinary Drugs Control (Beijing, China) and preserved by the National Engineering Laboratory of Bio-feed Safety and Pollution Prevention (Hangzhou, China). This strain was confirmed to be positive for *E. coli* K88 or *E. coli* K12 as determined by PCR genotyping. The bacteria were grown aerobically at 37°C in Luria-Bertani (LB) broth. Bacteria were diluted in PBS to a final concentration of approximately 1 × 10^9^ colony-forming units (CFU)/mL [4].

### Animals, challenge procedures and sample collection

Animal protocols were approved by the animal care committee of Zhejiang University in accordance with the Guide for the Care and Use of Agricultural Animals in Research and Teaching. A total of 12 weaned piglets (Duroc × Landrace) were treated with vancomycin (1 g/kg of diets) and streptomycin (5 g/kg of diets) for 3 d to reduce the normal levels of commensal flora. Then they were randomly assigned to two treatments with 6 piglets per group. Challenged piglets were orally administered with 100 mL of the prepared *E. coli* K88 suspension at a dose equivalent to 10^10^ CFU/pig every other 24 h for 3 d [27]. Control animals received 100 mL of sterile solution containing 10% (w/v) NaHCO_3_ and 20% (w/v) sucrose. At the end of the experiment, all piglets were narcotized and slaughtered. Tissues from liver, spleen, kidney, duodenum, ileum, jejunum and colon were collected and immediately frozen in liquid nitrogen. Blood samples were stored in coagulating tubes and centrifuged at 1,000 g at 4°C for 30 min to obtain the serum. The samples were stored at −80°C until further analysis. Colonic and cecal faeces were collected, homogenized and used to quantitate the colonization of *E. coli* on selective eosin methylene blue agar plates.

### Serum iron, total iron binding capacity, and tissue iron

Concentrations of serum iron and total iron binding capacity (TIBC) were measured with commercial kits (Jiancheng, China) following the manufacturer’s instructions. Transferrin saturation is the value of serum iron divided by the TIBC. Liver and spleen tissue samples were dried and their non-heme iron content was performed using BIOCHEMTEST as described previously [28].

### Histopathology

HE staining for duodenum and jejunum was performed to determine the extent of tissue damage as previously described [28]. Duodenum and jejunum were removed and placed overnight in fixative containing 10% formalin. Samples were then paraffin-embedded and cut at 5 μm in the longitudinal plane. Sections were examined under the microscope and compared with the control group.

### Quantitative real-time PCR and western blot analysis

Total RNA was isolated with Trizol reagent. Complementary DNA (cDNA) was synthesized using a reverse transcription kit. PCR was performed using a SYBR Premix Ex *Taq*^™^ kit on a Step One Plus^™^ real-time PCR System (Applied Biosystems, Carlsbad, CA, USA). The gene-specific primers are presented in Table 1. Messenger RNA (mRNA) expression levels were determined using the 2^-ΔΔ*Ct*^ method [29] with porcine 18S rRNA or bacterial 16S rRNA as a reference.

**Table 1:** Primer sequences for the real-time PCR amplification

mRNA	Primer sequence (5′-3′)
18S	F: CCCACGGAATCGAGAAAGAG
	R: TTGACGGAAGGGCACCA
TNF-α	F: CCAATGGCAGAGTGGGTATG
	R: TGAAGAGGACCTGGGAGTAG
IL-1β	F: ACAAAAGCCCGTCTTCCTG R: ATGTGGACCTCTGGGTATGG
IFN-γ	R: CAAAGCCATCAGTGAACTCATCA F: TCTCTGGCCTTGGAACATAGTCT
Ferritin-H	F: CAGCCTCCCGCCATGAC R: TGACATGGACAGGTAGACGTAGGA
Hepcidin	F: GAGCCACCGCTGGTTTGAC R: ACATCCCACAGATTGCTTTGC
DMT1	F: CGCTTCGCCCGAGTGAT
	R: TGAGATGCTCTACATCCTGGAAGAC
FPN	F: GAATAATGGGAACTGTGG R: AAGTGGCTCTGTCTGAAT
TFRC	F: AACCCAGCAGAAGCATTGTCTT R: TTAATATAAGTGAAAGCCTTTAAATGCAA
TFR2	F: TGGAGTTCTACTTCCTGTCCCAGTAC R: AGCGTGTGGTCTTCCGTGAC
IL-6	F: TGGCTACTGCCTTCCCTACC R: CAGAGATTTTGCCGAGGATG
HFE	F: GGTGATCCTGGGCTGTGAA
	R: CCCATCGTACCCATACTTCCA
HJV	F: ACGTCTGGAAACACTCATTCTCAA R: AAGGCTCAGGGTGGAAGATACA
HO-1	F: ATGCCCCAGGATTTGTCAGA R: CCATCACCAGCTTAAAGCCTTCT
IL-17	F: CAAGAACTTCCCTCAGCATGTA
	R: GAGGTGAAGCGTTTGGAGTAA
IL-22	F: AAGGGTGATGACCAGCATATC
	R: CAGGGCCATAAACAGCAAATAC
Lcn2	F: AGCCAGTTCGCCATAGTATTC
	R: AGCGGACAAAGTTCTCCTTC
16S	F: CCTTACGACCAGGGCTAC R: GACTACGACGCACTTTATGAG
bfr	F: TTATGCCGATAGCGTTCA R: GTTCCGTTTCCAGCCAGT
ftnA	F: GACCAATCAGGACTACCCAACA R: CAGACCTTCGCCGCTTTT
fepA	F: CACCTGGTTCCGTAACGATTAT R: GCACGTTATCCCACTGATAGAG
entS	F: GGCACGTTTATTACCTTGCTAC R: ATTTCAACGGATGCTCACG
entD	F: GCCGTGGTATCTCGTCAA R: TCGTGTTCCGCTGGTGTA

The concentration of protein in extracts from tissues or cells was determined using a BCA Protein Assay Kit. Extracts containing equal quantities of proteins (50 μg) were resolved on polyacrylamide gels and transferred to PVDF membranes. The membranes were blocked for nonspecific binding for 30 min (5% skimmed protein in PBS) and incubated overnight at 4°C with antibodies for FPN (Rabbit anti-FPN, Bioss, CHN), ferritin-H (Rabbit anti-ferritin-H, Bioss, CHN), Lipocalin 2 (Mouse anti-Lipocalin-2, Abcam) and β-actin (Mouse anti-β-actin, Huabio, CHN). Blots were developed using ECL detection reagents, exposed on Kodak Xdmat blue XB-1 film, and quantified by ImageJ software.

### Tissue culture assays

Intestinal porcine epithelial cell J1 (IPEC-1) cells were grown in a 1:1 mixture of DMEM/F12 and 10% fetal calf serum (GIBCO). To achieve polarization, cells were seeded on the apical compartment in a 24 mm diameter Transwell plate (Corning) and utilized when a transepithelial resistance of at least 1500 Ω.cm^2^ was reached.

### Stimulation with cytokines and bacteria infection

Recombinant porcine IL-17 and IL-22 were obtained from R&D Systems and utilized at the final concentration of 100 ng/mL, respectively. For *E. coli* K88 infection, 10^4^ CFU bacteria were added on the apical side of Transwell plate for 4 h.

### Bacterial growth in cell culture supernatant

*E. coli* K88 and *E. coli* K12 were grown overnight in iron-limiting conditions (LB supplemented with 0.2 mM 2,2-Dipyridyl, Sigma-Aldrich) at 37°C with aeration. Approximately 5,000 or 8,000 CFU were inoculated into the supernatant collected from the apical or basolateral side of the Transwell plate 24 h after stimulation with IL-17 and IL-22 or mock control. CFU were enumerated by plating serial dilution 5 h after inoculation. Recombinant human Lcn2 was obtained from R&D Systems and utilized at the final concentration of 0.1, 0.5 and 1.0 µg/mL, respectively.

### Bacteria treatment with FeCl_3_ or 2,2-Dipyridyl

*E. coli* K12 at mid logarithmic phase was transferred to 5 mL LB media in the presence of FeCl_3_ (50 μM, 100 µM) or 2,2-Dipyridyl (0.2 mM, Sigma-Aldrich). Cells were collected by centrifugation 0.5 h after incubation. Total RNA extractions were performed as described above.

### CAS assay for siderophore

CAS assay solutions were prepared as previously described [30]. The ternary complex chrome azurol S/iron (III)/hexadecyltrimethylammonium bromide serves as an indicator. When siderophores removed the iron from the dye, its color turns from blue to orange. Bacterial cells at mid logarithmic phase were collected by centrifugation and resuspended with sterile water. 10 μL suspension contained 10^6^ CFU bacteria was transferred to 5 mL M9 minimal medium (iron limited) with or without FeCl_3_. Sterile water was added for a negative control. Then the treated groups were shaked at 37°C overnight. The culture supernatant collected by centrifugation was mixed with CAS assay solutions at a ratio of 1:1 and incubated for 1 h. Finally, the absorbance of mixture was measured at OD_630_ for indicating the value of siderophore secretion.

### Statistical analysis

The differences between treatment groups were analyzed by ANOVA followed by a Student’s *t*-test. Data were presented as mean ± SEM. A *p* value equal to or below 0.05 was considered statistically significant.

## Abbreviations

Lcn2: lipocalin 2
*E. coli*: *Escherichia coli*
HJV: hemojuvelin
TFRC: transferrin receptor
FtH: ferritin-H
FPN: ferroportin
DMT1: iron importer divalent metal transporter 1
DP: 2,2-Dipyridyl
IL: interleukin
LB: Luria-Bertani
CFU: colony-forming units
HE: Hematoxylin and eosin staining
cDNA: complementary DNA
IPEC-1: Intestinal porcine epithelial cell J1
ADFI: average daily feed intake
ADG: average daily gain

## References

[1] Gozzelino R, Arosio P (2016). Iron Homeostasis in Health and Disease. Int J Mol Sci.

[2] Cassat JE, Skaar EP (2013). Iron in infection and immunity. Cell Host Microbe.

[3] Nairz M, Schroll A, Sonnweber T, Weiss G (2010). The struggle for iron - a metal at the host-pathogen interface. Cell Microbiol.

[4] Ewaschuk JB, Murdoch GK, Johnson IR, Madsen KL, Field CJ (2011). Glutamine supplementation improves intestinal barrier function in a weaned piglet model of Escherichia coli infection. Brit J Nutr.

[5] Skaar EP (2010). The battle for iron between bacterial pathogens and their vertebrate hosts. PLoS Pathog.

[6] Raymond KN, Dertz EA, Kim SS (2003). Enterobactin: an archetype for microbial iron transport. Proc Natl Acad Sci USA.

[7] Fischbach MA, Lin H, Liu DR, Walsh CT (2006). How pathogenic bacteria evade mammalian sabotage in the battle for iron. Nat Chem Biol.

[8] Cusumano CK, Hung CS, Chen SL, Hultgren SJ (2010). Virulence plasmid harbored by uropathogenic Escherichia coli functions in acute stages of pathogenesis. Infect Immun.

[9] Flo TH, Smith KD, Sato S, Rodriguez DJ, Holmes MA, Strong RK, Akira S, Aderem A (2004). Lipocalin 2 mediates an innate immune response to bacterial infection by sequestrating iron. Nature.

[10] Berger T, Togawa A, Duncan GS, Elia AJ, You-Ten A, Wakeham A, Fong HE, Cheung CC, Mak TW (2006). Lipocalin 2-deficient mice exhibit increased sensitivity to Escherichia coli infection but not to ischemia-reperfusion injury. Proc Natl Acad Sci USA.

[11] Nelson AL, Ratner AJ, Barasch J, Weiser JN (2007). Interleukin-8 secretion in response to aferric enterobactin is potentiated by siderocalin. Infect Immun.

[12] Bachman MA, Miller VL, Weiser JN (2009). Mucosal lipocalin 2 has pro-inflammatory and iron-sequestering effects in response to bacterial enterobactin. PLoS Pathog.

[13] Nweze EI (2010). Aetiology of diarrhoea and virulence properties of diarrhoeagenic Escherichia coli among patients and healthy subjects in southeast Nigeria. J Health Popul Nutr.

[14] Ren W, Chen S, Yin J, Duan J, Li T, Liu G, Feng Z, Tan B, Yin Y, Wu G (2014). Dietary Dietary arginine supplementation of mice alters the microbial population and activates intestinalinnate immunity. J Nutr.

[15] Tang Y, Li F, Tan B, Liu G, Kong X, Hardwidge PR, Yin Y (2014). Enterotoxigenic Escherichia coli infection induces intestinal epithelial cell autophagy. Vet Microbiol.

[16] Nabuurs MJ (1998). Weaning piglets as a model for studying pathophysiology of diarrhea. Vet Q.

[17] Ratledge C, Dover LG (2000). Iron metabolism in pathogenic bacteria. Annu Rev Microbiol.

[18] Ganz T (2011). Hepcidin and iron regulation, 10 years later. Blood.

[19] Nemeth E, Tuttle MS, Powelson J, Vaughn MB, Donovan A, Ward DM, Ganz T, Kaplan J (2004). Hepcidin regulates cellular iron efflux by binding to ferroportin and inducing its internalization. Science.

[20] Ong ST, Ho JZ, Ho B, Ding JL (2006). Iron-withholding strategy in innate immunity. Immunobiology.

[21] Harrison PM, Arosio P (1996). The ferritins: molecular properties, iron storage function and cellular regulation. Biochim Biophys Acta.

[22] Singh V, Yeoh BS, Xiao X, Kumar M, Bachman M, Borregaard N, Joe B, Vijay-Kumar M (2015). Interplay between enterobactin, myeloperoxidase and lipocalin 2 regulates E. coli survival in the inflamed gut. Nat Commun.

[23] Zheng Y, Valdez PA, Danilenko DM, Hu Y, Sa SM, Gong Q, Abbas AR, Modrusan Z, Ghilardi N, de Sauvage FJ, Ouyang W (2008). Interleukin-22 mediates early host defense against attaching and effacing bacterial pathogens. Nat Med.

[24] Monack DM (2014). The battle in the gut. Immunity.

[25] Behnsen J, Jellbauer S, Wong CP, Edwards RA, George MD, Ouyang W, Raffatellu M (2014). The cytokine IL-22 promotes pathogen colonization by suppressing related commensal bacteria. Immunity.

[26] Goetz DH, Holmes MA, Borregaard N, Bluhm ME, Raymond KN, Strong RK (2002). The neutrophil lipocalin NGAL is a bacteriostatic agent that interferes with siderophore-mediated iron acquisition. Mol Cell.

[27] Gao Y, Han F, Huang X, Rong Y, Yi H, Wang Y (2013). Changes in gut microbial populations, intestinal morphology, expression of tight junction proteins, and cytokine production between two pig breeds after challenge with Escherichia coli K88: A comparative study. J Anim Sci.

[28] Pu Y, Guo B, Liu D, Xiong H, Wang Y, Du H (2015). Iron Supplementation Attenuates the Inflammatory Status of Anemic Piglets by Regulating Hepcidin. Biol Trace Elem Res.

[29] Schmittgen TD, Livak KJ (2008). Analyzing real-time PCR data by the comparative C-T method. Nat Protoc.

[30] Schwyn B, Neilands JB (1987). Universal Chemical-Assay for the Detection and Determination of Siderophores. Anal Biochem.

